# Corpus Callosum Diffusion Restriction in Neonatal Congenital Myotonic Dystrophy

**DOI:** 10.7759/cureus.94776

**Published:** 2025-10-17

**Authors:** Nanoha Sakatani, Hiroyuki Ikeda, Yuki Inami, Yasushi Noguchi, Katsunori Fujii

**Affiliations:** 1 Department of Pediatrics, Japanese Red Cross Narita Hospital, Chiba, JPN; 2 Department of Pediatrics, International University of Health and Welfare Narita Hospital, Chiba, JPN

**Keywords:** corpus callosum, diffusion magnetic resonance imaging, myotonic dystrophy, newborn infant, respiratory insufficiency

## Abstract

Congenital myotonic dystrophy (CDM) is the most severe form of myotonic dystrophy type 1 (DM1), typically presenting in the neonatal period with hypotonia, respiratory distress, and feeding difficulties. Neuroimaging findings in neonates with CDM are rarely described. We present a genetically confirmed case of CDM in a neonate who demonstrated restricted diffusion in the genu and splenium of the corpus callosum (CC). The patient developed respiratory failure at three weeks of age and required mechanical ventilation. The brain MRI performed 11 days after onset revealed restricted diffusion in the genu and splenium of the CC. The follow-up imaging at four months showed resolution of diffusion restriction and mild atrophy of the genu. Genetic testing confirmed CDM with 1000-1150 trinucleotide (CTG) repeats. We considered that the diffusion restriction observed in this patient may have been evidence of hypoxic-ischemic encephalopathy associated with slowly progressive respiratory failure in CDM.

## Introduction

Congenital myotonic dystrophy (CDM) is the most severe form of myotonic dystrophy type 1 (DM1), typically presenting in the neonatal period with hypotonia, respiratory distress, and feeding difficulties [1-3]. While the clinical features of CDM are well described, neonatal neuroimaging findings remain underreported [4-6]. Here, we report a neonate with genetically confirmed CDM who exhibited restricted diffusion in the genu and splenium of the corpus callosum (CC), suggestive of pre-Wallerian degeneration (pWD) due to hypoxic ischemic encephalopathy.

## Case presentation

**Figure 1:**
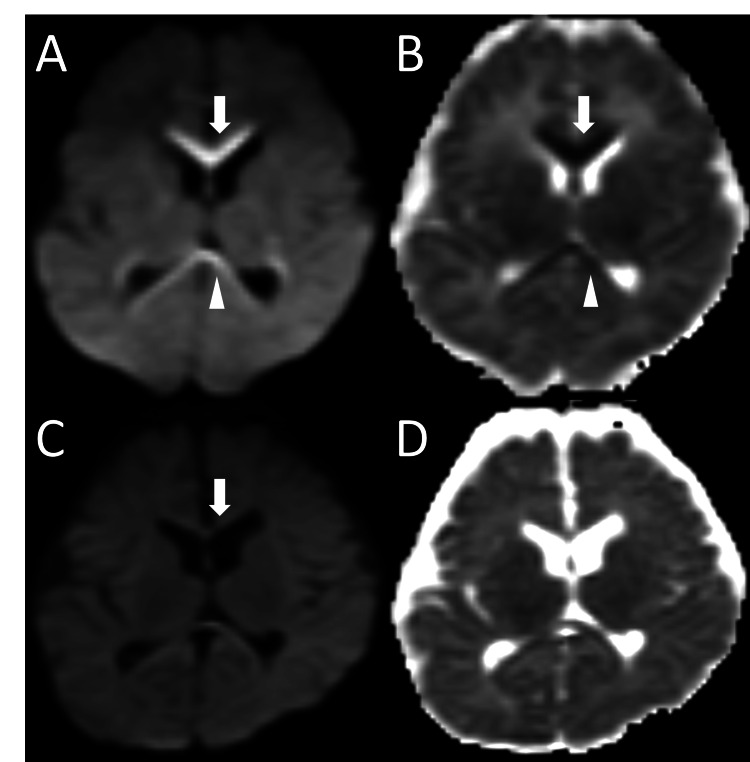
Diffusion restriction in the corpus callosum in congenital myotonic dystrophy Axial diffusion-weighted imaging (DWI) and apparent diffusion coefficient (ADC) maps are shown at the level of the corpus callosum. (A, B) MRI obtained at 32 days of age (hospital day 10) demonstrates high signal intensity in the genu (arrow) and splenium (arrowhead) on DWI (A), with corresponding low signal on the ADC map (B), indicating restricted diffusion. (C, D). A follow-up MRI at four months shows resolution of the previously observed diffusion restriction and mild atrophy of the genu of the corpus callosum (arrow).

A female infant was born at 38 weeks and four days of gestation via spontaneous vaginal delivery, weighing 2710 g. There was no polyhydramnios or concern regarding fetal movements during pregnancy. She cried promptly after birth without signs of neonatal asphyxia. The Apgar score was not available. She was discharged on day five without any apparent abnormalities. However, since she developed pallor, irregular breathing, and poor feeding, she was admitted to our hospital on day 23. On admission, she was lethargic, cyanotic, hypothermic, and tachycardic, with episodes of apnea. Her muscle tone was normal, but her cry had been weak for the three days preceding admission. Oxygen saturation was 85%, frequently dropping below 70%. We immediately treated her with emergency ventilation, intubation, and intensive care unit admission. Her weight was 3100 g, reflecting an average daily weight gain of approximately 25 g. Although initial laboratory tests and microbiological studies were unremarkable, venous blood gas analysis revealed respiratory and metabolic acidosis (Table 1). The results of all the newborn mass screening tests, including tandem mass spectrometry, were negative, providing additional clinical context. Imaging (chest X-ray, echocardiography, and head computed tomography) showed no abnormalities. However, the brain MRI on hospital day 10 (age 32 days) revealed restricted diffusion in the genu and splenium of the CC (Figures 1A, 1B) and a small lesion in the left periventricular white matter (data not shown). These areas appeared hyperintense on the diffusion-weighted imaging (DWI) and showed low values on the apparent diffusion coefficient maps, with no corresponding abnormalities on the T1- or T2-weighted images. Her respiratory status gradually improved, allowing extubation on hospital day eight, and she was discharged on day 15 without complications.

**Table 1:** Results of laboratory tests Venous blood gas analysis, performed 30 minutes after initiation of emergency ventilation, showed evidence of both respiratory and metabolic acidosis. The most critical abnormal values are highlighted in bold. pH: Potential of hydrogen, pCO₂: Partial pressure of carbon dioxide, HCO₃: Bicarbonate hydrogen carbonate, Na: Sodium, K: Potassium, Cl: Chloride

Test	Result	Reference value (for age & gender)	Unit
Venous blood gas			
pH	7.235	7.31–7.41	
pCO_2_	51.5	40–50	mmHg
HCO_3_^-^	21	23–28	mmol/L
Base excess	-7	-2–2	mmol/L
Glucose	117	70–105	mg/dL
Lactate	7.5	0.5–2.2	mmol/L
Hematology			
White blood cells	16100	4800–18500	/μL
Hemoglobin	20	11–15	g/dL
Platelet	465	150–450	x 10^3^/μL
Biochemistry			
Total bilirubin	0.7	0.4–3.2	mg/dL
Aspartate aminotransferase	26	20–62	U/L
Alanine aminotransferase	18	11–45	U/L
Lactate dehydrogenase	293	198–404	U/L
Alkaline phosphatase	557	530–1610	U/L
Urea nitrogen	14	3.7–15.5	mg/dL
Creatinine	0.21	0.3–0.8	mg/dL
Na^+^	130	135–143	mmol/L
K^+^	5.8	4.1–6.0	mmol/L
Cl^-^	97	101–111	mmol/L
Creatine kinase	74	44–310	U/L
C-reactive protein	0.17	0–0.3	mg/dL
Cerebrospinal fluid			
Cells	4	0–20	/μL
Mononuclear cells	91	60–80	%
Polymorphonuclear cell	9	20–40	%
Protein	45	30–100	mg/dL
Glucose	59	40–80	mg/dL

The follow-up MRI at four months showed resolution of the previously observed diffusion restriction and mild atrophy of the genu of the CC (Figures 1C, 1D). Development was reported as normal until age three years, but she showed mild intellectual disability at age six years. At age 10 years, the patient was diagnosed with CDM after her mother was diagnosed with DM1 due to muscle weakness. Genetic testing revealed 1000-1150 CTG repeats in the patient and 200 in the mother.

## Discussion

In this report, we observed diffusion restriction in the genu and splenium of the corpus callosum, associated with progressive respiratory failure in a child with CDM. These lesions disappeared after four months, leaving behind atrophy, suggesting that they were due to hypoxic-ischemic encephalopathy caused by respiratory failure in CDM.

MRI, particularly diffusion-weighted imaging (DWI), can detect early brain injury in neonates [7]. One such process is Wallerian degeneration (WD), in which axons and their myelin sheaths degenerate distal to the site of injury, typically over days to weeks following hypoxia or trauma [8]. A preceding stage, known as pre-Wallerian degeneration (pWD), involves early axonal changes, such as swelling and cytoskeletal disruption, that appear as restricted diffusion on MRI before structural abnormalities become apparent [9,10]. The pWD has been mainly reported in severe hypoxic-ischemic encephalopathy (HIE), affecting pathways such as the corticospinal tracts and the corpus callosum (CC) [9,10].

Previously reported neuroimaging findings in CDM include ventriculomegaly and periventricular white matter abnormalities; however, CC involvement has been rarely described [4-6]. In our case, restricted diffusion in the genu and splenium of the CC likely represents pWD, supported by several observations. First, the 11-day interval between respiratory compromise and MRI is consistent with the reported timeframe for pWD following hypoxic-ischemic injury in neonates, which typically ranges from one to two weeks [9,10]. Second, mild genu atrophy observed on follow-up imaging suggests residual structural change secondary to earlier axonal injury. Third, although restricted diffusion in the splenium can occasionally be an incidental finding in infants under four months, particularly on 3T MRI, this is unlikely in our patient because a 1.5T scanner was used, where the reported prevalence is very low (~3%) [11].

The small lesion observed in the left periventricular white matter may also reflect focal hypoxic-ischemic injury, possibly occurring in parallel with the axonal changes in the CC. This finding is consistent with earlier reports describing periventricular white matter involvement in CDM [4-6] and may indicate that both focal and tract-specific injuries can occur in the neonatal period.

The differential diagnosis included mild encephalitis/encephalopathy with a reversible splenial lesion (MERS), which can present with transient splenial diffusion restriction [12,13]. However, in neonates, incomplete myelination reduces the likelihood of intramyelinic edema, the presumed mechanism of MERS [12]. Other causes, such as viral infections, including human parechovirus, metabolic disorders, or postictal changes, were not supported by clinical history or laboratory findings. Taken together, these considerations support the interpretation that the observed CC lesions might reflect pWD in the context of CDM complicated by hypoxic-ischemic insult.

This report has some limitations. CDM results from large CTG repeat expansions in the 3′ untranslated region of the myotonic dystrophy protein kinase gene, causing multisystem involvement that includes both skeletal muscle and the central nervous system [1-3]. In particular, the immature white matter tracts in CDM may exhibit intrinsic vulnerability due to abnormal axonal development, potentially predisposing the CC to injury even in the absence of severe hypoxia [4,6]. Nevertheless, the neonatal presentation often features profound hypotonia and respiratory insufficiency, both of which increase susceptibility to hypoxic events. In this case, the observed brain changes may reflect an interplay between intrinsic vulnerability of white matter tracts and secondary hypoxic-ischemic injury; however, the possibility that the lesions were purely hypoxia-related cannot be excluded, as detailed neonatal observations were not available.

## Conclusions

This case suggests that restricted diffusion in the genu and splenium of the CC may represent early axonal injury in neonates with congenital myotonic dystrophy, potentially reflecting both intrinsic vulnerability of white matter tracts and secondary hypoxic-ischemic injury in the setting of respiratory compromise. Although not definitive, MRI findings such as these could provide early imaging evidence of central nervous system involvement in CDM. Further studies are warranted to clarify their diagnostic value and long-term clinical implications.

## References

[1] Echenne B, Rideau A, Roubertie A (2008). Myotonic dystrophy type I in childhood Long-term evolution in patients surviving the neonatal period. Eur J Paediatr Neurol.

[2] Campbell C, Levin S, Siu VM (2013). Congenital myotonic dystrophy: Canadian population-based surveillance study. J Pediatr.

[3] Zapata-Aldana E, Ceballos-Sáenz D, Hicks R, Campbell C (2018). Prenatal, neonatal, and early childhood features in congenital myotonic dystrophy. J Neuromuscul Dis.

[4] Kuo HC, Hsiao KM, Chen CJ (2005). Brain magnetic resonance image changes in a family with congenital and classic myotonic dystrophy. Brain Dev.

[5] Romeo V, Pegoraro E, Ferrati C (2010). Brain involvement in myotonic dystrophies: neuroimaging and neuropsychological comparative study in DM1 and DM2. J Neurol.

[6] Peglar LM, Nagaraj UD, Tian C, Venkatesan C (2019). White matter lesions detected by magnetic resonance imaging in neonates and children with congenital myotonic dystrophy. Pediatr Neurol.

[7] Robertson RL, Glasier CM (2007). Diffusion-weighted imaging of the brain in infants and children. Pediatr Radiol.

[8] Ota K, Nakazato Y, Seo K (2025). Clinical and magnetic resonance imaging features in acute ischemic stroke with early wallerian degeneration: a case-control study. BMC Neurol.

[9] Bekiesinska-Figatowska M, Duczkowska A, Szkudlinska-Pawlak S (2017). Diffusion restriction in the corticospinal tracts and the corpus callosum in neonates after cerebral insult. Brain Dev.

[10] Hayakawa K, Tanda K, Nishimura A (2022). Diffusion restriction in the corticospinal tract and the corpus callosum of term neonates with hypoxic-ischemic encephalopathy. Pediatr Radiol.

[11] Tan AP, Lim YT (2019). “Restricted diffusion” within the splenium of the corpus callosum: a potential pitfall in young infants on 3T imaging and marker of normal myelin maturation. Neuropediatrics.

[12] Tada H, Takanashi J, Barkovich AJ (2004). Clinically mild encephalitis/encephalopathy with a reversible splenial lesion. Neurology.

[13] Tetsuka S (2019). Reversible lesion in the splenium of the corpus callosum. Brain Behav.

